# Public awareness, attitude, and practice regarding food labeling, Alexandria, Egypt

**DOI:** 10.1186/s40795-023-00770-5

**Published:** 2024-01-19

**Authors:** Azza Mehanna, Ayat Ashour, Doaa Tawfik Mohamed

**Affiliations:** 1 Health Education and Behavioral Sciences, Department of Health Administration and Behavioral Sciences, High Institute of Public Health, Alexandria University, Alexandria, Egypt; 2 Department of Family Health, High Institute of Public Health, Alexandria University, Alexandria, Egypt; 3 Department of Nutrition, High Institute of Public Health, Alexandria University, Alexandria, Egypt

**Keywords:** Food labeling, Awareness, Attitude, Consumer rights, Egypt, Alexandria Governorate, Cross sectional study

## Abstract

**Background:**

Food labeling is an important public health tool allowing consumers to make informed and healthy choices. Considering how important it is for consumers to be aware of food labels while choosing healthy foods, this study aims at assessing public awareness, attitude, and practice regarding food labeling in Alexandria Governorate, Egypt.

**Methods:**

A cross sectional study using a predesigned interview questionnaire to collect data from 719 adult consumers from both sexes (28.2% males: 71.8% females), recruited from different branches of one of the largest supermarkets in Alexandria.

**Results:**

More than half of the participants (55.6%) reported reading food labels. The most frequently read information was production/ expiry date (76.8%), product name (68.3%), and ingredients (38.0%) while the least frequently read was nutritional facts (29.9%). More than half of the study sample (50.9%) had low awareness about the content of food labels, nevertheless, having higher scores on awareness about food labels predicted reading practice. Nearly three-quarters of the respondents had average to good food label reading practice. Most respondents (81.8%) had a favorable attitude about food labeling in its present form, however, higher attitude scores did not seem to influence their use of food labels. Being older (β = 0.045, CI; 0.014–0.076, p = 0.005), being female (β = 1.162, CI; 0.541–1.784, p = 0.000), having secondary education or equivalent (β = 1.042,CI; 0.050–2.034, p = 0.040), having university education or beyond (β = 3.090, CI; 2.132–4.048, p = 0.000), and having higher scores on awareness about food labels (β = 1.407, CI; 1.324–1.490, p = 0.000) were significant predictors of reading food labels.

**Conclusions:**

Most of the studied consumers had a positive attitude towards food labeling, however, more than half of them had low awareness of food labeling content. Nutritional facts were the least frequently read by consumers. Reading food labels was significantly predicted by having higher education, being older, being a female, and having better awareness.

**Supplementary Information:**

The online version contains supplementary material available at 10.1186/s40795-023-00770-5.

## Background

The promotion of healthy eating and lifestyles is one of the most difficult and complex objectives that public health is working to achieve on a global scale [1]. The burden of nutrition-related chronic diseases including overweight, obesity, cardiovascular diseases, diabetes, and cancer is increasing in the Eastern Mediterranean Region [2]. The risk factors for these diseases are closely related to food consumption, dietary patterns, nutrition, and lifestyles [3]. The World Health Organization developed a regional strategy on nutrition 2010–2019 addressing issues of over nutrition to overcome the increasing rates of obesity and diet-related non-communicable diseases. One of its important activities was to establish food labelling regulations and to improve products labelling especially for products containing high fat, sugar, and salt [4].

Food labeling is a major tool that enables people to get information about the food they purchase and make nutritionally appropriate choices [5, 6]. It provides information on the composition, ingredients, and their proportional amounts as well as on the quality, origin, processing, and preservation [7]. “Labeling includes any written, printed, or graphic matter present on the label that accompanies the food, or is displayed near the food” [8].

Labeling guidelines are adopted in Egyptian standard 1546/2011. On January 2017, the Egyptian Parliament approved law number 1/2017 establishing the Egyptian National Food Safety Authority (NFSA). One of the NFSA functions is to develop protocols and rules for the mandatory food labeling criteria [9].

Nutrition facts tables (nutrition information panels) found on the back or side of food packages, comprise a list of nutrients and their amounts. Nutrition facts panels are health promoting tools that guide people in selecting and purchasing food products. Egypt is adopting voluntary nutrition labeling regulations unless when a nutrient (or health) claim is made and for foods with special dietary uses (e.g. infant formula, cereal based food for young children) [10]. These regulations are following the guidelines from the Codex Alimentarius Commission (CAC/GL 2-1985 revised 1993 and 2011) [11].

A systematic review of 120 studies found that self-reported use of nutrition label was typically greater than 50% [12]. The actual use of nutrition labels is quite low, and certain groups are more likely to do so, particularly women, those with higher incomes and/or higher educational levels, and those who are already interested in diet and health. There have also been reports of widespread difficulty in comprehending and interpreting the somewhat complex numerical information on the back-of-pack nutrition label [12–14].

There has been inconsistency in the definition of food labels use where most global studies refer to it as checking nutrition labels [5, 6]. Moreover, there has been little research found on Arab consumers’ use of food labels. A review of the literature on the use of food labels by Arab consumers found that the major conclusions back up suggestions for nutrition education and awareness campaigns meant to raise food label literacy and usage frequency among Arab consumers [15].

On the local level in Egypt; no similar studies have been found except for two small studies; the first was conducted in Menoufiya Governorate, which assessed the knowledge, attitude, and practice of mothers attending family units as regards nutritional label of canned food [16]. The other one discussed the role of food labels in selection and handling of packed food and its microbiological evaluation among the staff and personnel of Alexandria University [17]. In this context, and due to the public health importance of reading information on food labels on helping consumers recognize their food choices, this research was conducted aiming at assessing public awareness, attitude, and practice regarding food labeling in Alexandria Governorate, Egypt.

## Methods

**Study aims**:

- To detect consumers’ awareness about the content of food labels.
- To detect consumers’ attitude towards the utility, clarity, and comprehensibility of food label in its current form.
- To determine the predictors of consumers’ usage of food labels.
- To identify the relationship between consumers’ awareness of certain food additives and their willingness to purchase food items containing one or more of these additives.
- **Study design**: A cross sectional study design was used to conduct the study.
- **Study setting**: The study was conducted in different branches of one of the largest supermarkets in Alexandria Governorate, Egypt.

### Study participants

Adults aged ≥ 18 years of both sexes, who could read and write and attended the mentioned setting for shopping in the period of the field work and accepted to participate in the study. Exclusion criteria: Those who could not communicate verbally and non-Egyptians.

### Sample size

The minimum required sample size was estimated to be 700 at 5% degree of precision, α = 0.05 and design effect = 2, where the prevalence of awareness regarding food labelling was assumed to be 65% [18]. Sample size was calculated using EPI-info version 7 software.

### Sampling method

Cluster sampling technique was used. Alexandria consists of eight districts, four of them were randomly selected (Al-Montazah, Sharq, Wasat, and El Gomrok districts) and from each selected district one branch of the supermarket was randomly selected [19, 20]. The total sample size was distributed equally on the 4 branches. Participants were included consecutively until the sample size was fulfilled **(n = 719)**.

### Data collection

Data was collected by four groups of trained data collectors covering the four selected districts over a period of three months. Data was collected from each attendee using the following tool:

1. A pre- designed pre-coded structured interview questionnaire was constructed by the researchers based on previous literature (Additional file 1). The questionnaire was used to collect the following data:

- Personal data and sociodemographic characteristics: age, sex, marital status, having children, income, education, occupation, and presence of chronic diseases such as hypertension and diabetes.
- Public awareness about the content of current food labels including production/expiry date, list of ingredients, nutritional facts, and country of origin. A list of items (nine items) of food labels was presented to the respondents who were asked to indicate which of these items was included in food labels. The score ranged from 0 to 9, higher scores indicated higher awareness. The score was converted to percentage and categorized, according to Bloom’s classification, [21] into low awareness (< 50%), average awareness (50-80%), optimal awareness (> 80%).
- Public attitude towards current food labeling was assessed using a 3-item scale (disagree = 0, not sure = 1, agree = 2), inquiring whether current food labels were informative, useful, clearly written, and easy to understand and whether it would be preferable to use distinctive colors for healthy and unhealthy elements. The score ranged from 0 to 10, higher scores implied favorable attitude. The score was converted to percentage and was classified into unfavorable/negative (< 33%), neutral (33.3-66.6%) and positive/favorable (> 66.6%).
- The practice of reading food labels was assessed by asking participants to indicate how frequently they read each of the listed items (9 items) of food labels. The items were scored on a frequency rating scale with never = 0, sometimes = 1, always = 2. The total score ranged from 0 to 18, it was converted to percentage and classified into poor (< 33.3%), average (33.3-66.6%), good (> 66.6%).
- The frequency of reading the list of ingredients and/or nutrition facts was measured using a single statement rated on a frequency rating scale ranging from “never” to “always”. Participants’ reasons for reading/not reading the list of ingredients and/or nutrition facts were investigated. A list of possible causes of reading and not reading the list of ingredients and/or nutrition facts was prepared, and participants were asked to indicate the reasons relevant to them.
- Awareness of respondents about some food additives and its relation to their willingness to purchase the packaged food item containing these additives were assessed. A list of food additives (five) was presented to participants, they were asked to indicate whether they recognized each of these items and if the presence of any of them influenced their willingness to purchase the packaged food product (increased, decreased, no change).

A pilot study was carried out to determine face validity and to test the feasibility and comprehensibility of the questionnaire. Minor modifications were made accordingly. Cronbach’s α values were used as a measure of internal consistency, the calculated Cronbach’s α of the knowledge, attitude and practice sections was 0.914 and the Cronbach’s α of the whole questionnaire was 0.756.

### Ethical considerations

Approval of Ethics Committee of the High Institute of Public Health, Alexandria University was obtained. The approval of the manager of the administrative department of the supermarket in Alexandria was obtained. All methods were carried out in accordance with relevant guidelines and regulations. Informed consent was obtained from the participants after explaining the aim of the study. Confidentiality of the collected data of the participants was considered. No private questions were included. No obligation of any kind was used to let participants participate in the study, and any participant was free to withdraw from completing the study at any time. There was no conflict of interest.

### Statistical analysis

Collected data was revised, coded, and fed to statistical software IBM SPSS version 25 (SPSS, Inc. Chicago, IL). All statistical analysis was done using two-tailed tests. P-values less than 0.05 were considered statistically significant. Continuous variables were presented as mean and standard deviation, while categorical variables were presented as frequencies and percentages. Multiple linear regression analysis was used to control for confounding factors and investigate significant predictors of participants’ food label reading practice [22].

## Results

The mean age of participants was 37.2 ± 11.0 years, 71.8% of participants were females, 68.3% were married, 90.7% had children and 47.4% were university graduate or beyond. Half of the participants (50.5%) were working, the monthly income of about two-thirds (64%) was just enough and 19.3% of the study sample reported having chronic diseases (Table 1).

**Table 1:** Socio-demographic and personal characteristics of the studied sample

Socio-demographic and personal characteristics	Total (n = 719)	
	No.	%
**Age (years)**		
≤ 30	238	33.1
31–50	397	55.2
51->60	84	11.7
**Mean ± SD**	37.2 ± 11.0	
**Gender**		
Males	203	28.2
Females	516	71.8
**Marital status**		
Single	183	25.5
Married	491	68.3
Divorced	31	4.3
Widowed	14	1.9
**Education**		
Read and write	58	8.1
Primary/preparatory	126	17.5
Secondary or equivalent	194	27.0
University graduate or beyond	341	47.4
**Having children**		
No	233	32.4
Yes	486	67.6
**Income**		
Not enough	212	29.5
Just enough	460	64.0
Enough and saving	47	6.5
**Occupation**		
Working	363	50.5
Not Working	345	48.0
Retired	11	1.5
**Presence of chronic diseases**		
No	580	80.7
Yes^#^	139	19.3

# Hypertension, DM, Cancer, renal, hepatic, bone, and thyroid diseases

More than half of the study sample (50.9%) had low awareness about the content of food labels such as production/expiry date, list of ingredients, nutritional facts, and country of origin (Table 2).

**Table 2:** Awareness about the content of food labels

Awareness score	Total (n = 719)	
	No.	%
Low	366	50.9
Average	148	20.6
Optimal	205	28.5
**Mean ± SD**	4.85 ± 3.05	

One quarter of the participants (25.5%) had poor scores concerning reading food labels (Table 3).

**Table 3:** Overall food label reading practice

Food label reading practice score	Total (n = 719)	
	No.	%
Poor	183	25.5
Average	277	38.5
Good	259	36.0
**Mean ± SD**	9.88 ± 5.72	

**Fig. 1:**
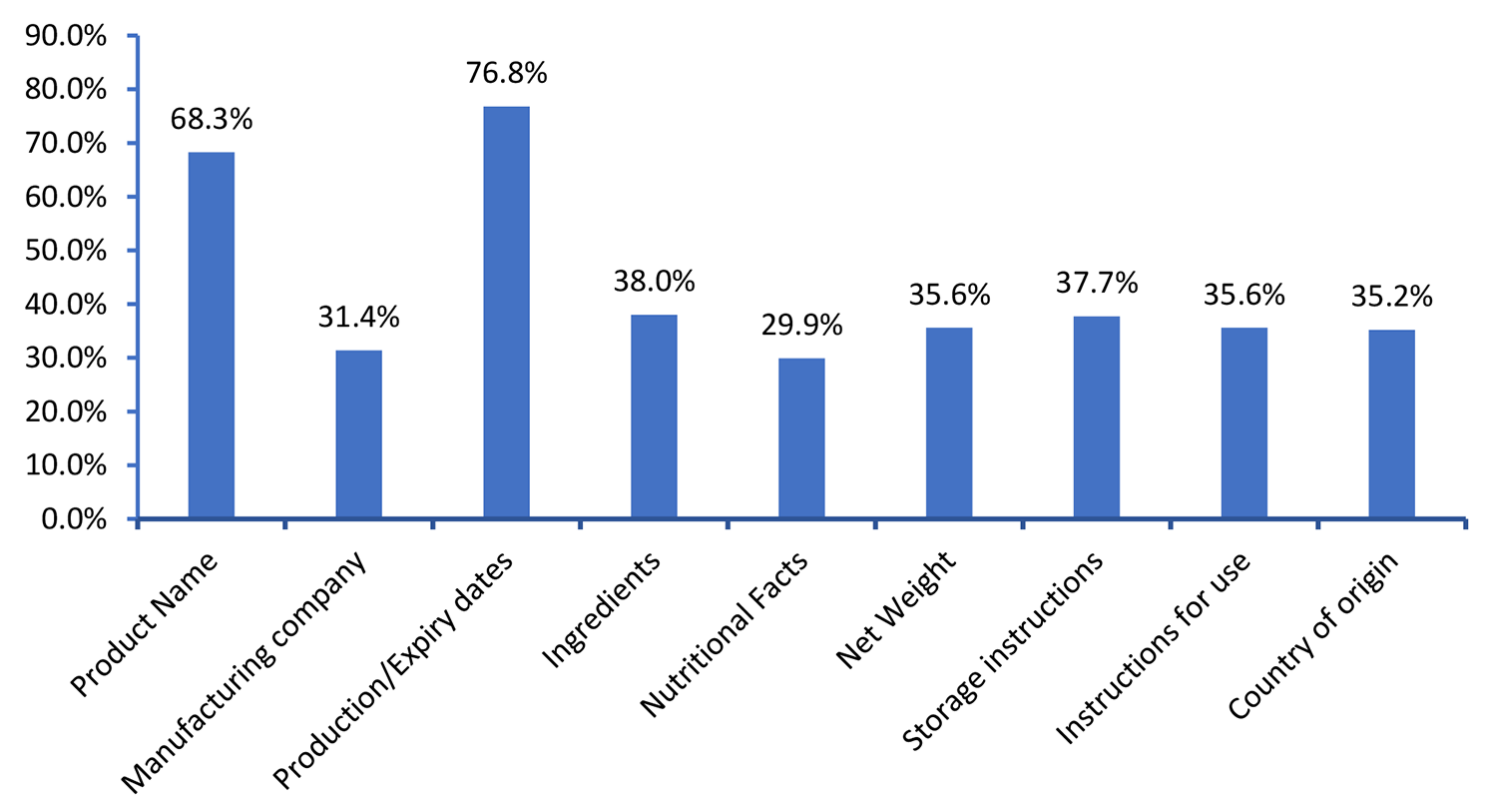
Food label content items read by the participants (%)

Production/ expiry date, product name, and ingredients seemed to be the most frequently read labels (76.8%, 68.3%, 38.0%, respectively), while nutritional facts were the least frequently read (29.9%) (Fig. 1).

**Fig. 2:**
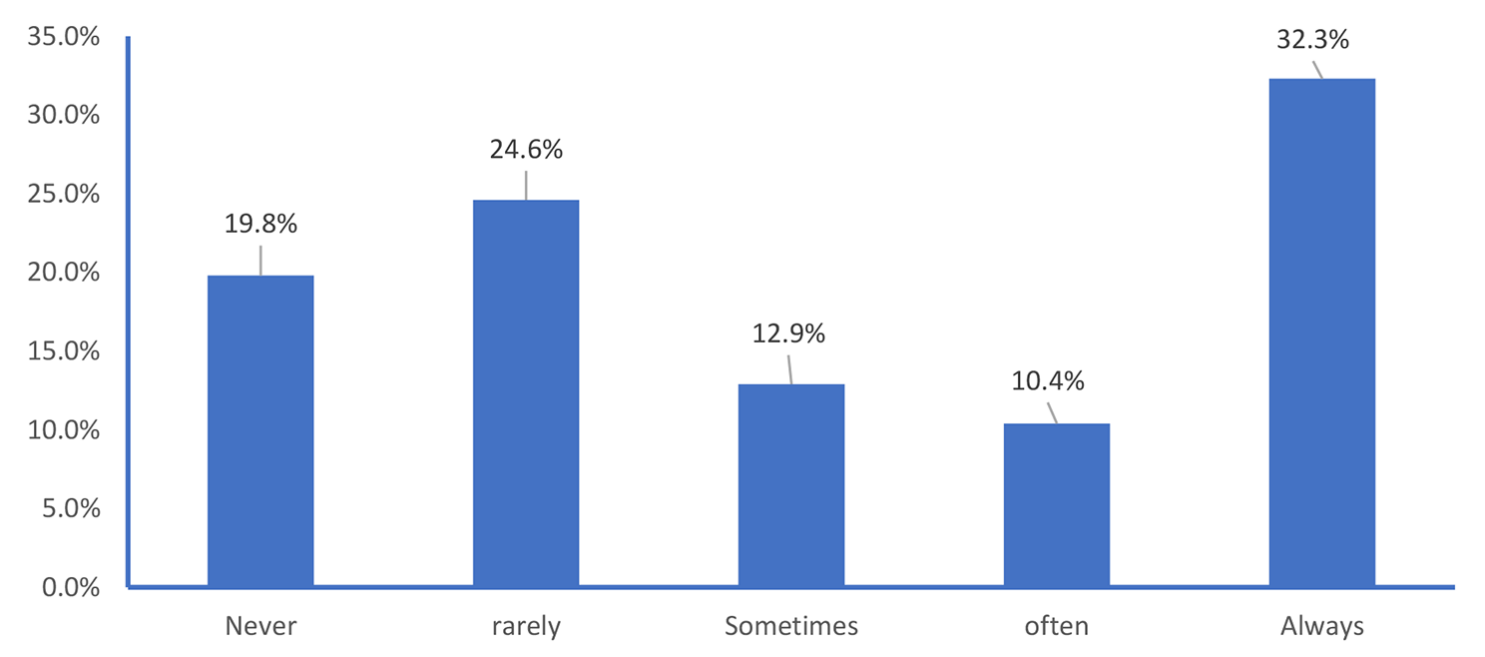
Frequency of reading food ingredients and/or nutrition facts (%)

Regarding frequency of reading the list of ingredients and/or nutrition facts, 44.4% of customers reported never or rarely reading it while it was always read by 32.3%, often read by 10.4% and sometimes read by 12.9% (Fig. 2).

Among customers who reported reading the list of ingredients and/or nutrition facts (55.6%), the most frequently mentioned causes were to avoid fat rich food (50.3%), to avoid food-related diseases such as cancers, diabetes, and cardiac diseases (47.3%), to avoid food rich in sugar (40.5%) and to avoid food rich in salt (34.8%). While the least mentioned causes were to avoid/choose food rich in proteins and/or fibers (2.5%, 1.5% respectively). Among those who reported not reading (44.4%), the highest rated cause was “don’t care” (49.8%) followed by “lack of time” (28.2%), then “small font size” (22.6%) and “don’t trust the information” (18.5%), (Fig. 3a and b).

**Fig. 3a:**
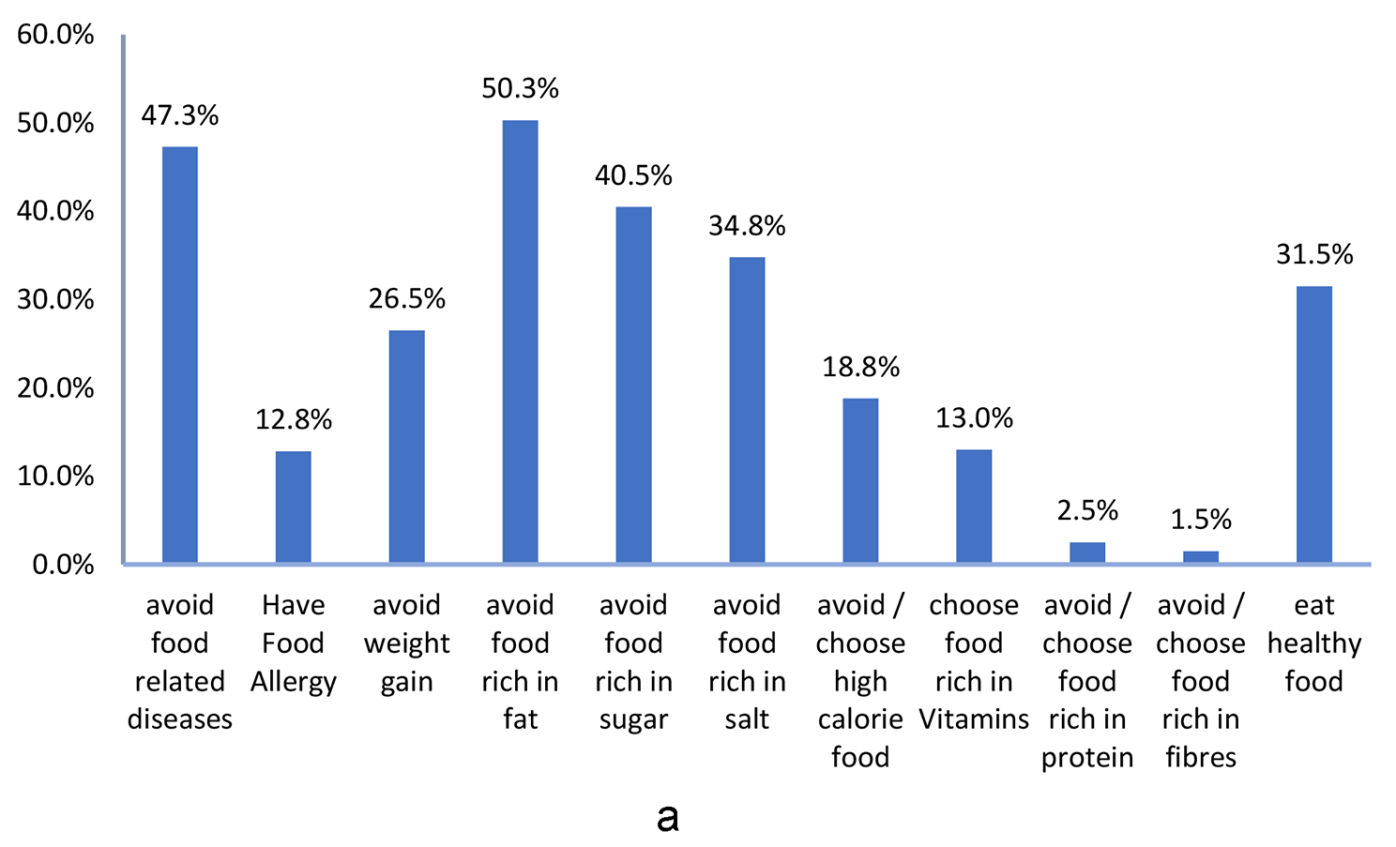
Causes of reading list of ingredients and/or nutrition facts *. *Multiple response variable

**Fig. 3b:**
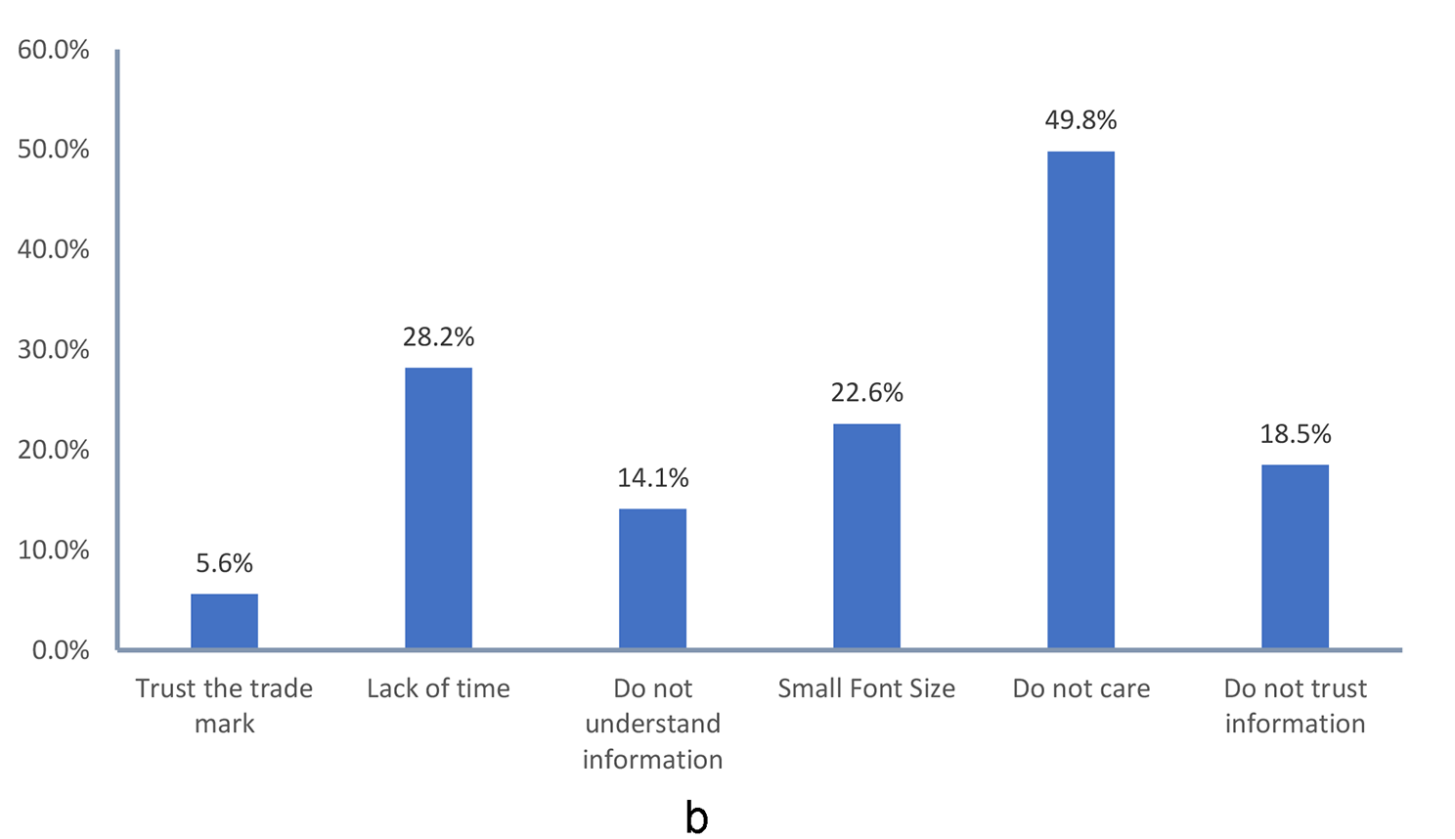
Causes of not reading list of ingredients and/or nutrition facts*. *Multiple response variable

Most of the study participants showed favorable attitude towards current food labeling in Egypt (81.8%), while 17.1% were undecided and only 0.7% had unfavorable attitude (Table 4).

**Table 4:** Overall attitude towards the current food label form

Attitude Score	Total (n = 719)	
	No.	%
Negative (unfavorable)	5	0.7
Neutral	126	17.5
Positive (favorable)	588	81.8
**Mean ± SD**	7.166 ± 1.027	

**Fig. 4:**
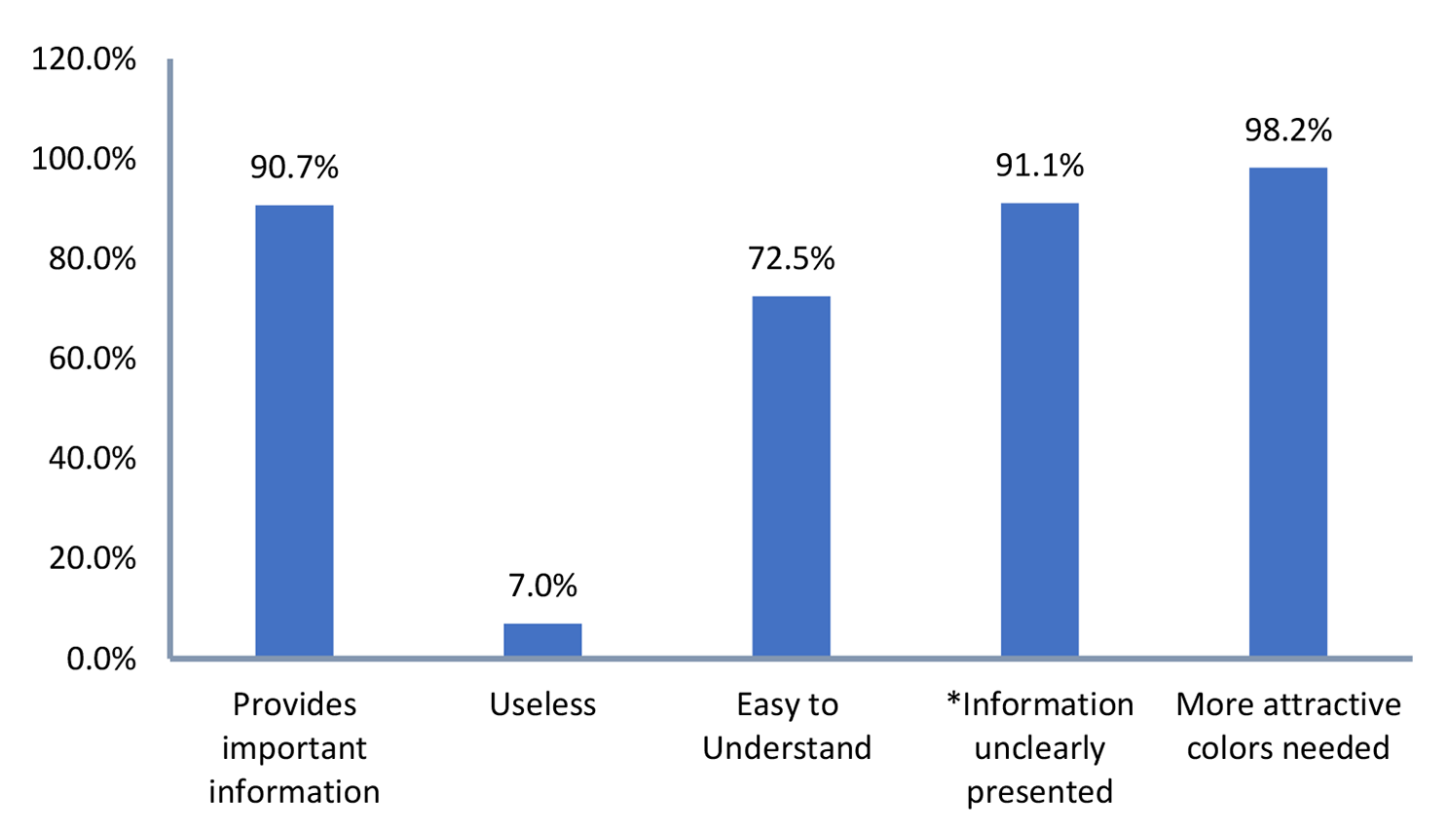
Percent of agreement with each item of the attitude scale. *Letters very small or wiped out

Most of the studied customers acknowledged the importance (90.7%), usefulness (93%) and comprehensibility (72.5%) of food labeling. However, almost all the study participants (98.2%) agreed that more attractive colors needed to be used and more than 90% agreed that the information was not clearly presented (e.g., letters very small or wiped out) (Fig. 4).

After adjustment for all possible confounders, it was found that the significant predictors of reading food labels were being older (β = 0.045, CI; 0.014–0.076, p = 0.005), being female (β = 1.162, CI; 0.541–1.784, p = 0.000), having secondary education or equivalent (β = 1.042,CI; 0.050–2.034, p = 0.040), having university education or beyond (β = 3.090, CI; 2.132–4.048, p = 0.000), and having higher scores on awareness about food labels (β = 1.407, CI; 1.324–1.490, p = 0.000). These factors explained 68% of the variance in reading food label practice (R^2^ = 0.683) (Table 5).

**Table 5:** Multiple linear regression analysis for variables affecting participants’ food label reading practice

Predictors	Unstandardized Coefficients (B)	Significant	95.0% Confidence Interval for B		
			LL		UL
**Age** (years)	0.045	0.005*	0.014	0.076	
**Sex** (female)	1.162	0.000*	0.541	1.784	
**Presence of chronic diseases** (yes)	0.636	0.073	-0.059	1.332	
**Marital status**					
Married	0.044	0.935	-1.014	1.102	
Divorced/widowed	0.561	0.437	-0.857	1.980	
**Income**					
Just enough	0.490	0.080	-0.059	1.039	
Enough and saving	-0.501	0.346	-1.543	0.542	
**Education**					
Primary/preparatory	0.656	0.205	-0.359	1.670	
Secondary or equivalent	1.042	0.040*	0.050	2.034	
University graduate or beyond	3.090	0.000*	2.132	4.048	
**Occupation** (working)	-0.053	0.846	-0.588	0.482	
**Having children** (Yes)	0.196	0.687	-0.758	1.150	
**Total attitude score**	0.058	0.625	-0.176	0.292	
**Total awareness score**	1.407	0.000*	1.324	1.490	

Adjusted linear regression model; F = 111.698, p = 0.00, adjusted R^2^ = 0.683

*Significant variable p < 0.05

Added sugars ranked first among the known food additives by the customers (40.9%) followed by sodium nitrate (30%) then hydrogenated oils (29.2%) and palm oil (21.7%), whereas monosodium glutamate was the least known (5.8%) (Table 6).

**Table 6:** Awareness of participants about some food additives

Food Additives	Total (n = 719)	
	No.	%
Sodium nitrate	216	30.0
Added sugars	294	40.9
Aspartame	106	14.7
Monosodium glutamate	42	5.8
Palm oil	156	21.7
Hydrogenated oils	210	29.2

**Fig. 5:**
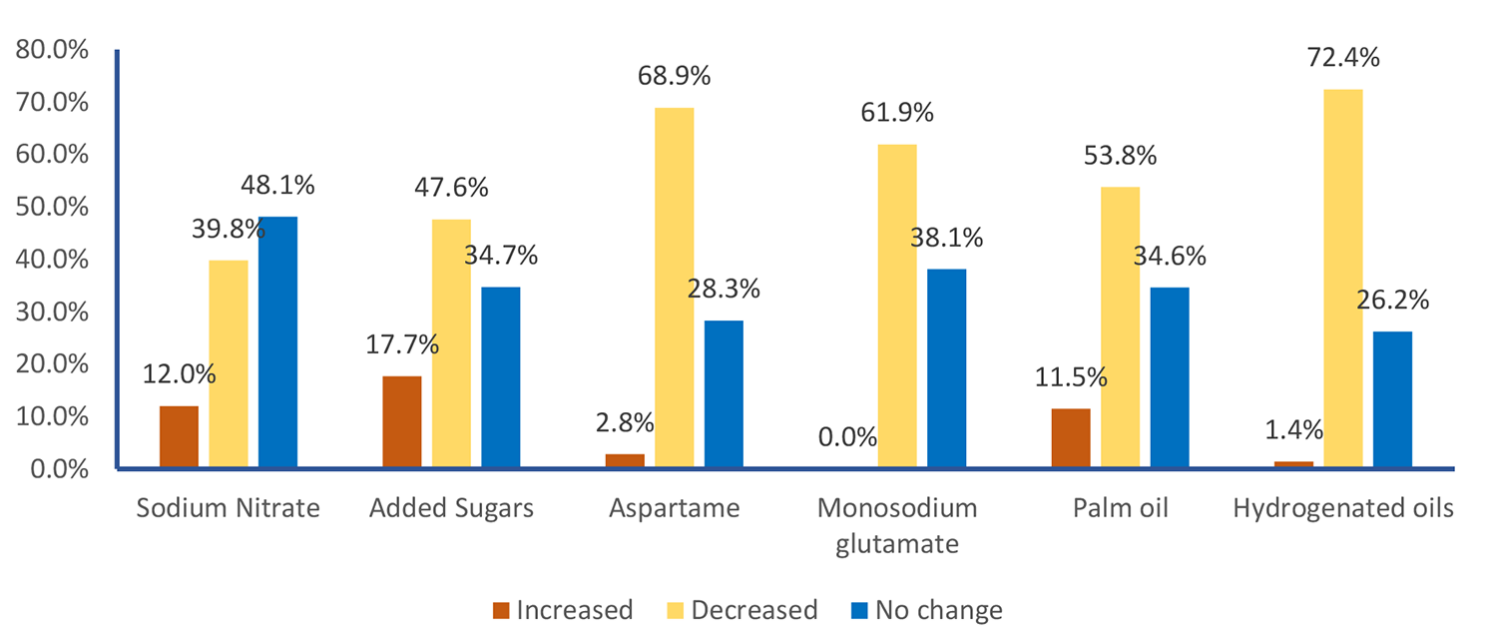
Willingness of participants to buy additive-containing food items

A great percentage of the study participants who were aware of these additives would choose not to buy food products on recognizing their presence among food ingredients; hydrogenated oils (72.4%), aspartame (68.9%), monosodium glutamate (61.9%), palm oil (53.8%) and added sugars (47.6%). However, about half of them would not change their decision about buying a sodium nitrate- containing food product (48.1%) and more than one-third would still be willing to purchase products containing monosodium glutamate (38.1%) and added sugars (34.7%) (Fig. 5).

## Discussion

Poor nutrition, unhealthy, and unbalanced diets due to global modernization are closely related to the alarming rise of the burden of overweight, obesity and consequent chronic diseases [23, 24]. Dietary guidelines encourage consuming nutrient-dense foods and beverages with the least amounts of added sugars, saturated fat, and sodium [25].

Food labeling is a major tool that enables people to get information about the food they purchase and make nutritionally appropriate choices [18]. Food labeling regulations have been established to improve product labelling especially for products containing high fat, sugar, and salt [4].

Consumers’ awareness, attitudes, and use of food labeling are mostly related to the educational level, interest, trust or understandability of the information on the label or first-time purchasing [26, 27].

In the present study, it was found that most of the consumers were females, probably because purchasing foods for the household is considered one of the gender roles of the female in Egypt.

Most previous studies looked at nutrition or health information on food labels, [5, 6, 12] however, the present study broadened to include any information on the food labels. The current study revealed that more than half of the participants (55.6%) reported reading food labels. Similarly, a survey conducted among US adult consumers reported that 59% of them read labels (mostly nutrition facts and ingredients) before buying packaged food products [28]. However, far lower frequencies were reported by other studies. For instance; in Arab United Emirates, most of the respondents (89.6%) read food labels and in Portugal, 65.7% usually read the information on food product labels [18, 29].

The most frequently read information by the present study participants was production/ expiry date (76.8%), product name (68.3%), and ingredients (38.0%). Similar findings were reported by other studies, [18, 30, 31] which indicates that consumers are usually concerned with the harm that may result from eating expired and unknown products and, to some extent, keen to explore the cooking ingredients. Despite the growing awareness of food safety and nutrition-health interplay, the nutritional facts were of least (29.9%) concern for the study participants. Consistently, a study in Indore city in India observed that only 9.3% of the participants use nutrition information when shopping [30]. Likely, only 27% of UK shoppers looked at nutrition information on the label according to an observational research carried out in- stores [32]. A Portuguese study found that 41.2% reported non checking the nutritional value of the food products they bought [29]. On the contrary, around 70% of consumers in both Australia and New Zealand reported using the nutrition information panel and the ingredients statement [31]. The low interest of the public to read the nutritional facts may reflect their negative attitude towards the nutritional facts section; they may find it useless, difficult to understand, or not clearly written (letters are very small or wiped out for example). In the current study, despite the agreement of most participants on the importance and usefulness of food labels, most of them reported that they needed to be more clearly presented and more colorful.

In a similar vein, on assessing the attitude of consumers in 16 European countries about food labeling, the majority did not show a favorable attitude due to the use of technical and numerical information on labels [29]. Consistent findings were reported by Seyedhamzeh et al. [33] The findings of the present study revealed that most respondents had a favorable attitude about food labeling in its present form, however, high attitude scores did not seem to influence their use of food labels.

Scientists maintain that an individual’s attitude influences his/her behavior, [34] nevertheless, in the present study, it seems that having higher awareness about food labels and being in fact, able to read and understand the presented information were more effective in reading food labels than just perceiving them useful and important.

Most studies on the use of food labels found a significant relation between knowledge and use. Knowledge about nutrition can be expected to affect understanding and use of label [32, 35]. Findings of the present study added support to previous literature where, having higher scores on awareness about food labels significantly predicted reading food labels.

Sociodemographic factors (e.g., age, education) were reported by several studies [18, 29, 32, 36, 37] to significantly influence the search for, use and understanding of labeling information. This is consistent with the present study results; where being older, being a female, and having secondary education or beyond were significant predictors for reading food labels.

Respondents who read the list of ingredients and/or nutrition facts mentioned some causes for doing so which included avoiding fat rich food (50.3%), avoiding food-related diseases such as cancers, diabetes, and cardiac diseases (47.3%), avoiding food rich in sugar (40.5%), and avoiding food rich in salt. In accordance, other studies reported that participants checked labels when a member of the family was ill; had diabetes or heart problem (75.2%) or wanted to buy healthier food (46.2%) [29, 33]. On the other hand, non-readers of the list of ingredients and/or nutrition facts reported different reasons including no interest, lack of time, and small font size of the information on the label.

In the current study, participants were asked if they were aware about certain food additives such as added sugars, transfat, monosodium glutamate, aspartame, sodium nitrate (salt) and palm oil. The frequency of unaware participants exceeded that of ones for each of the listed elements. Expectedly, added sugars (40.9%) and sodium nitrate (30%) were the most recognized elements while monosodium glutamate was the least known additive (5.8%). We found that sizable proportions of the customers who were aware about hydrogenated fat (*transfat*) and palm oil would not be willing to purchase packaged food items containing these additives (72.4% and 53.8% respectively). This is consonant with the findings of Seyedhamze et al. [33] who reported that fat content was one of the most important reasons for reducing consumption or not choosing certain food products. Moreover, 47.6% and 68.9% of the study participants were aware about added sugars or aspartame respectively would not choose to buy food products on recognizing the presence of these additives. Similarly, Australian consumers were found to link their decision of buying a product to its sugar content and type [31].

The study has some limitations. First, because the study was cross-sectional, a temporal relationship between exposure and outcome cannot be established. Moreover, the tool used for collecting data was an interview questionnaire, thus data might have been subjected to information or social desirability biases.

### Conclusion and recommendations

Generally, most of the studied customers had a positive attitude towards food labeling as they found it important and useful. However, more than half of them had low awareness of food labelling content. Despite its health-related importance “Nutritional facts” was the least frequently read by customers mostly because they did not care, did not have time or due to small font size. Evidently, awareness about food labeling has a significant influence on using them. Another seemingly important factor is the display of food labeling including font, color, and clarity. Using food labels was significantly predicted by having higher education, being older, being a female, and having better awareness. Food labeling plays an essential role in making appropriate nutritional choices. Hence, this study sheds light on the need for raising awareness and enhancing the knowledge of public about food labels and nutrition facts. Moreover, the food industry should be made aware of the importance of proper display of nutrition information on packaged food.

### Practical implications

Raising public awareness would benefit from the traditional and contemporary media. Designing campaigns to underscore the importance of reading food labels and giving information about the different food label items with the help of MOHP in Egypt would make an important step in raising public awareness. Campaigns can make use of broadcast media such as television and radio and print media as posters and pamphlets. However, nowadays, social media have a significant influence on public awareness hence, specialists can design websites, pages, and advertisements to draw public attention to food labelling and its association with their nutritional choices and their health.

### Electronic supplementary material

Below is the link to the electronic supplementary material.

Supplementary Material 1

## Data Availability

The datasets used and/or analyzed during the current study are available from the corresponding author on reasonable request.
