# Supplementary material for: Public awareness, attitude, and practice regarding food labeling, Alexandria, Egypt
Source: BMC Nutr. 2024 Jan 19;10:15. doi: 10.1186/s40795-023-00770-5 (PMC10797718; doi:10.1186/s40795-023-00770-5)
Supplement: Supplementary file 1 — Supplementary Material 1 [file 40795_2023_770_MOESM1_ESM.pdf]

ALEXANDRIA UNIVERSITY
High Institute of Public Health
المعهد العالي للصحة العامة

Dear Participant

The aim of conducting this research study is to assess public awareness, attitude, and practice regarding food labeling in Alexandria Governorate, Egypt.

If you accept to participate in the study, please complete the following questionnaire.

Please be assured that all collected information will be confidential and used for research purposes only.

Thank you for your cooperation.

Public awareness, attitude, and practice regarding food labeling, Alexandria, Egypt

Questionnaire

<u>Socio-demographic data:</u>

1. Age:

2. Gender: (1) Male (2) Female

3. Marital status: (1) Single (2) Married (3) Divorced (4) Widow/widower

4. Number of children:

5. Education: (1) Illiterate/ read and write (2) Primary (3) Preparatory

(4) Secondary (5) University

6. Occupation: (1) No (2) Yes

7. Income: (1) Not enough (2) Just enough (3) Enough and saves

8. Chronic diseases: (1) Hypertension (2) Diabetes mellitus (3) Cancer
(4) Liver insufficiency (5) Renal insufficiency (6) Others ….. (7) No

The following question assesses your awareness of the current food labels present on packaged food. Please give all the possible answers you know.

9. What are the food labels present on packaged food?

0.Don't know
1.Name of the product
2. Name of food manufacturer (brand name)
3. Production/ Expiry date
4. List of ingredients
5. Nutrition facts
6. Net quantity
7. Special storage instructions
8. Instructions for use (when necessary).
9. Source of food/ country of origin
10. Others ……..

The following questions assess your attitude towards food labelling present on packaged food. Please choose the answer that best expresses your attitude.

10. Which of the following statements best describes your opinion about food labelling in general:

1. No need for food labelling
2. Only certain food items should be labeled (such as food for young children)
3. All food products should be labeled

How much do you agree with each of the following statements regarding current food labelling in Egypt:

**Statement:**	**(0) Disagree**	**(1) Not sure**	**(2) Agree**
11. Current food labelling is very informative			
12. Current food labelling is useless			
13. Current food labelling is easy to understand			
14. Some information is not clearly written or written in very small letters such as the manufacturing and expiry dates.			
15. It would be more useful to use front of pack labels that are more colorful, attractive and easy to understand			

The following questions assess your practice of reading food labels and their effect on your decision to purchase various packaged food items. Please choose the answer that best suits you.

What is the kind of information you look for on food labels?

**Information**	**(0) Never**	**(1) Sometimes**	**(2) Always**
16. Name of the product			
17. Name of food manufacturer (brand name)			
18.Production/ Expiry date			
19. List of ingredients			
20. Nutrition facts			
21. Net quantity			
22. Special storage instructions			
23. Instructions for use (when necessary).			
24. Source of food/ country of origin			

25. How often do you read the List of ingredients and/or nutrition facts (e.g., sugar, fat and salt content) before purchasing a food product for the first time?

(0) Never (1) Rarely (2) Sometimes (3) Often (4) Always

26. Reasons for reading list of ingredients and/or nutrition facts before purchasing a food product for the first time?

1. Avoid diseases related to food
2. Having food allergy/intolerance
3. Avoid gaining weight
4. Avoid high fat food
5. Avoid high sugar food
6. Avoid high salt food
7. Avoid/select high calorie food
8. Choose vitamin and mineral- rich food
9. Avoid/select protein- rich food
10. Avoid/select fiber-rich food
11. Choose more healthy food

27. Reasons for not reading list of ingredients and/or nutrition facts before purchasing a food product for the first time?

1. Brand loyalty
2. Lack of time
3. Lack of understanding = (Complicated presentation of information)
4. Small fonts make it difficult to be read
5. Lack of interest
6. Lack of trust in food label

The following substances are sometimes present in packaged food. Do you recognize any of these substances?

Substance	(0) No	(1) Yes
28.Sodium nitrate		
29.Added sugars		
30. Aspartame		
31. Monosodium glutamate		
32. Palm oil		
33. Hydrogenated oils		

How does the presence of any of the recognized substances on a packaged food item make you decide to (willingness to) purchase this food item?

Substance	(1) Increased willingness	(2) Decreased willingness	(3) Not changed
34. Sodium nitrate			
35. Added sugars			
36. Aspartame			
37. Monosodium glutamate			
38. Palm oil			
39. Hydrogenated oils			
